# Late-onset popliteal artery pseudoaneurysm secondary to implant migration in a pediatric patient: a case report and surgical management

**DOI:** 10.3389/fped.2025.1560952

**Published:** 2025-05-23

**Authors:** Senanur Duymaz, Burak Duymaz, Onur Gursan, M. Can Kosay

**Affiliations:** ^1^ Department of Cardiovascular Surgery, Dokuz Eylul University Faculty of Medicine, Izmir, Türkiye; ^2^ Department of Orthopedics and Traumatology, Dokuz Eylul University Faculty of Medicine, Izmir, Türkiye

**Keywords:** popliteal artery pseudoaneurysm, pediatric orthopedic complications, implant migration, autologous saphenous vein graft in pseudoaneurysm repair, vascular injury

## Abstract

**Introduction:**

Popliteal artery pseudoaneurysms are rare but potentially life-threatening complications following orthopedic procedures, particularly osteotomies. These pseudoaneurysms, characterized by a hematoma caused by disruption of the arterial wall and contained by surrounding tissues rather than all arterial layers, can cause severe ischemic damage if not promptly diagnosed and treated. Early detection and timely surgical intervention are critical to prevent catastrophic outcomes such as thrombosis, embolization, or limb loss. This case highlights the challenges in diagnosing and managing a delayed popliteal artery pseudoaneurysm caused by implant migration in a pediatric patient.

**Case Presentation:**

A 15-year-old male patient with cerebral palsy presented with sudden, severe knee pain and localized swelling around the knee joint. These symptoms occurred two years after he underwent a distal femoral extension osteotomy with lateral plate fixation. Initial imaging studies were inconclusive, suggesting possibilities such as soft tissue sarcoma or hematoma. However, an incisional biopsy unexpectedly revealed a pseudoaneurysm cavity adjacent to the popliteal artery. Further imaging identified that the distal screws of the femoral plate had migrated posteriorly due to femoral deformity, causing direct irritation and injury to the artery.

**Surgical Intervention:**

An urgent multidisciplinary surgical approach was implemented. The procedure began with femoral artery clamping to control bleeding, followed by excision of the pseudoaneurysm cavity. A saphenous vein graft was harvested and interposed to repair the damaged segment of the popliteal artery. To eliminate the source of irritation, the screws were removed. Postoperative ultrasonography confirmed successful arterial repair, with no signs of thrombosis or recurrence. The patient was mobilized early and discharged with an antiplatelet regimen, followed by regular clinical monitoring.

**Conclusion:**

This case emphasizes the importance of routine follow-up in pediatric patients with orthopedic implants, as skeletal growth and deformity progression can lead to implant migration and serious vascular complications. The delayed diagnosis underscores the need for heightened clinical suspicion and comprehensive imaging in patients with unusual postoperative symptoms. Early recognition and timely multidisciplinary surgical intervention are crucial for preventing life-threatening vascular injuries. This case also highlights the necessity of individualized implant removal planning in growing patients to avoid similar complications.

## Introduction

Popliteal artery pseudoaneurysms are rare but significant complications, often seen after distal femur osteotomy. These vascular injuries may arise from various mechanisms, including direct trauma to the artery during the surgical procedure or inadvertent manipulation of adjacent anatomical structures, which can compromise vascular integrity (1).

The significance of early diagnosis and timely intervention in medical scenarios is paramount, as any delays can precipitate serious complications, including thrombosis, embolism, or even limb ischemia. These conditions can result in profound and enduring repercussions for a patient's mobility and overall health status, as highlighted by Shin et al. (2). Specifically, when considering osteotomies performed around the knee joint, there is a notable association between the development of pseudoaneurysms and vascular trauma that may arise from surgical manipulation or inadvertent injury to the adjacent soft tissues. Such complications underscore the critical need for vigilant monitoring and swift action in the postoperative period to mitigate risks and safeguard patient outcomes. The intricate relationship between surgical technique and vascular integrity emphasizes the importance of skilled surgical practice and thorough preoperative planning to minimize the likelihood of these adverse events.

Research conducted by Szyber P Jr et al. sheds light on the occurrence of pseudoaneurysms in the popliteal artery, particularly as a complication following tibial osteotomy procedures (3). These vascular anomalies are frequently attributed to direct trauma to the arterial wall or injury to surrounding tissues that occurs during the surgical intervention. Such complications can manifest clinically in various ways, with patients often reporting localized pain and swelling in the aftermath of surgery. These symptoms can act as crucial warning signs for healthcare professionals, prompting them to investigate the potential for vascular complications that could arise from the surgical manipulation of the area.

In order to accurately diagnose popliteal artery pseudoaneurysms, healthcare providers typically rely on state-of-the-art imaging techniques. These advanced modalities may include ultrasound, computed tomography (CT) angiography, and magnetic resonance angiography (MRA). Each of these imaging options plays a pivotal role in providing detailed insights into the characteristics of the pseudoaneurysm, including its size, precise location, and the hemodynamic implications it may pose (4). Such diagnostic imaging is vital, as it enables clinicians to formulate appropriate management strategies and interventions tailored to the individual patient's condition, thereby optimizing outcomes and minimizing the risk of further complications. In addition to imaging, clinical assessment and patient history are crucial components that guide the decision-making process for treatment options.

When addressing the treatment of popliteal artery pseudoaneurysms, a range of options is available, contingent upon the size, characteristics, and overall clinical context of the pseudoaneurysm. In cases where there is substantial arterial injury, primary surgical repair of the affected artery is frequently the preferred intervention. This approach aims to restore the integrity of the arterial wall and ensure proper blood flow, thereby reducing the risk of complications.

In instances where the pseudoaneurysm presents as a larger defect or when there is significant damage to the arterial structure, autologous vein graft repair may be warranted. This technique involves using a segment of the patient's own vein to replace the damaged section of the artery, promoting better biocompatibility and reducing the risk of rejection that can occur with synthetic grafts (5).

A comprehensive and multidisciplinary approach is essential for optimizing patient outcomes in the management of popliteal artery pseudoaneurysms. A recent 10-year analysis highlights that prompt vascular consultation and early surgical intervention are key determinants of limb salvage in pediatric patients with traumatic vascular injuries (6). This collaborative model typically includes orthopedic surgeons, who may address associated musculoskeletal injuries; vascular surgeons, who specialize in the management of blood vessel disorders; and radiologists, who provide critical imaging support for accurate diagnosis and treatment planning. By leveraging the expertise of these diverse specialists, healthcare teams can effectively minimize the risk of long-term complications, such as limb ischemia or thrombosis, ensuring a more favorable prognosis for patients suffering from this vascular condition.

## Case report

**Figure 1:**
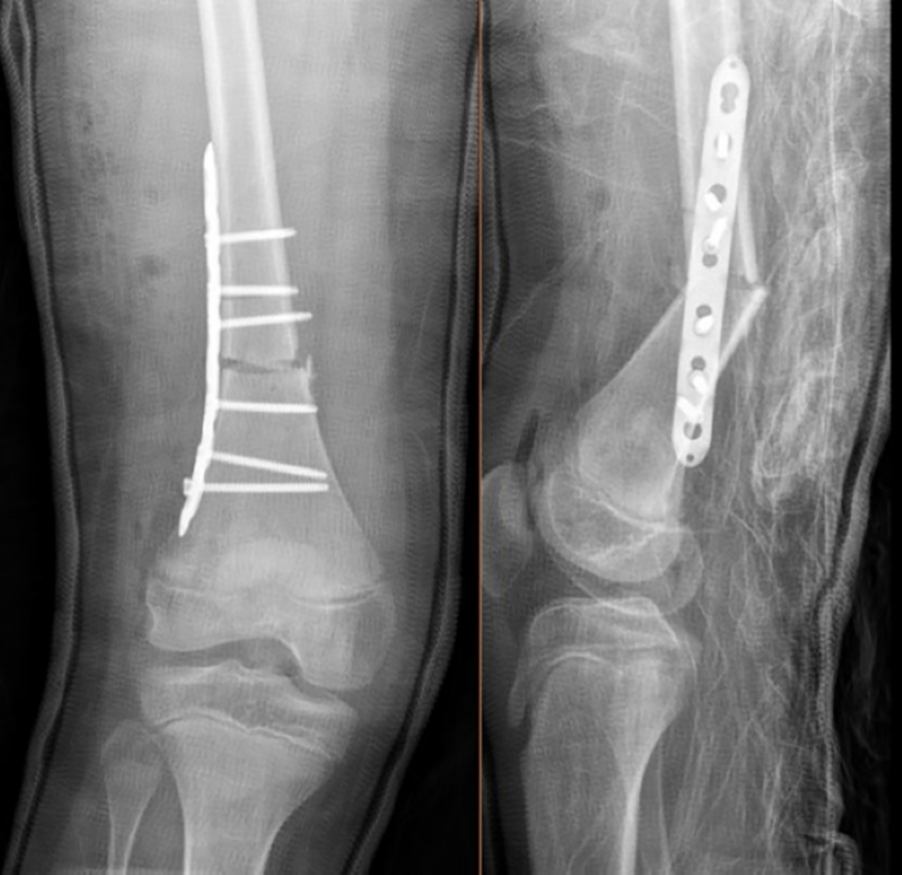
Early postoperative anteroposterior and lateral radiographs of the distal femur demonstrate proper alignment and fixation with a lateral femoral plate and screws. The screws are securely positioned within the femoral cortex without extending posteriorly, indicating no immediate risk of mechanical irritation or vascular compromise, particularly concerning the popliteal artery.

**Figure 2:**
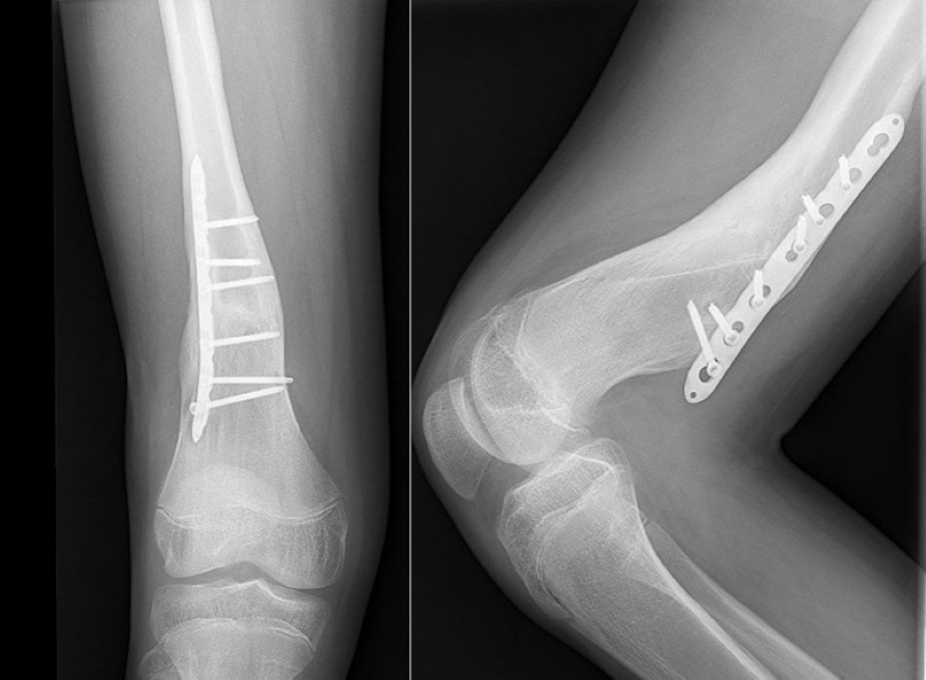
Late(2 year follow-up) postoperative knee x-ray demonstrating the extension of distal screws toward the posterior aspect of the femur due to implant migration. This finding indicates potential mechanical irritation to the popliteal artery.

A 15-year-old male patient, who has a well-documented medical history of cerebral palsy with a Gross Motor Function Classification System (GMFCS) level IV, presented with a sudden and acute onset of significant pain accompanied by noticeable swelling localized around the distal femur and the knee joint. These symptoms developed two years after he underwent a distal femoral extension osteotomy and distal femoral lateral plate osteosynthesis (Figures 1, 2). The index surgery was performed to increase knee extension, thereby facilitating easier caregiving and enhancing functional mobility. The patient had no prior history of vascular pathology, and no genetic or familial conditions were reported. He had been followed regularly by pediatric orthopedic and rehabilitation teams since early childhood. Prior to the osteotomy, he had only received conservative management including physical therapy; no previous surgical interventions had been undertaken.

Postoperatively, he was followed monthly for the first six months and then every six months. No complaints were noted during routine follow-ups, and his last clinical evaluation occurred approximately four months before symptom onset, at which time no abnormal findings were observed. The patient reported no progressive pain or discomfort prior to the event. Rather, he experienced a sudden onset of pain and swelling around the knee. At the external referring center, inspection revealed diffuse, circumferential swelling around the distal femur and knee joint, with no skin discoloration or pulsatility. On palpation, the mass was firm and tender, but non-pulsatile. Passive and active range of motion were severely limited due to pain and mechanical resistance from the mass. A neurovascular examination showed palpable distal pulses, and Doppler ultrasonography confirmed that peripheral perfusion was preserved, although vascular distortion was suspected. Given the unclear etiology and atypical presentation, the patient was referred to our center for further evaluation.

**Figure 3:**
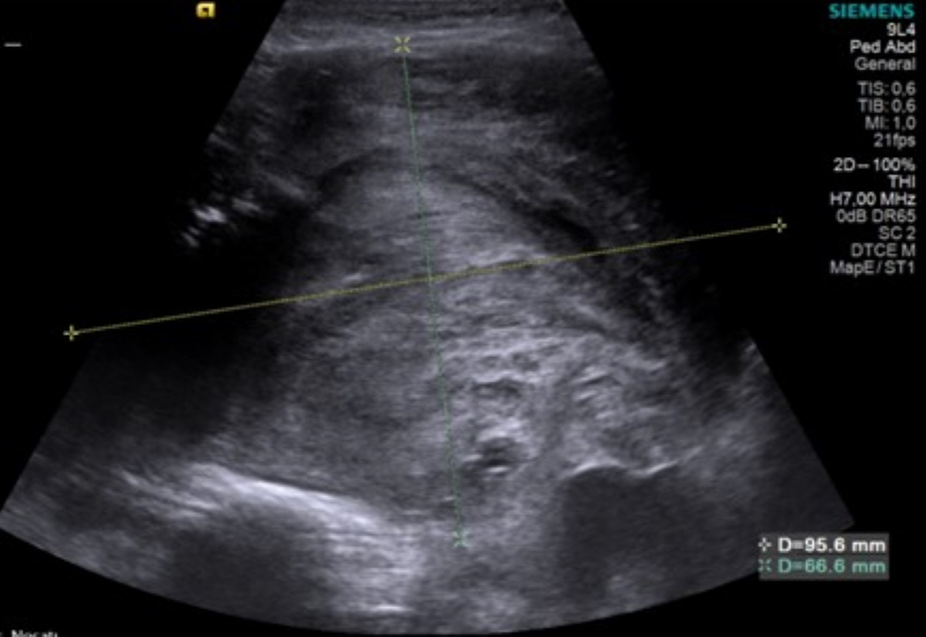
Late postoperative knee Doppler ultrasound image of the mass located in the distal one-third of the right thigh, extending anteromedially from the subcutaneous tissue through the muscle planes to the bone. The lesion measures approximately 9.5 × 11 × 6.5 mm, with a lobulated contour, heterogeneous internal structure, and solid-semisolid characteristics, including cystic components. No evidence of active arterial bleeding was observed.

**Figure 4:**
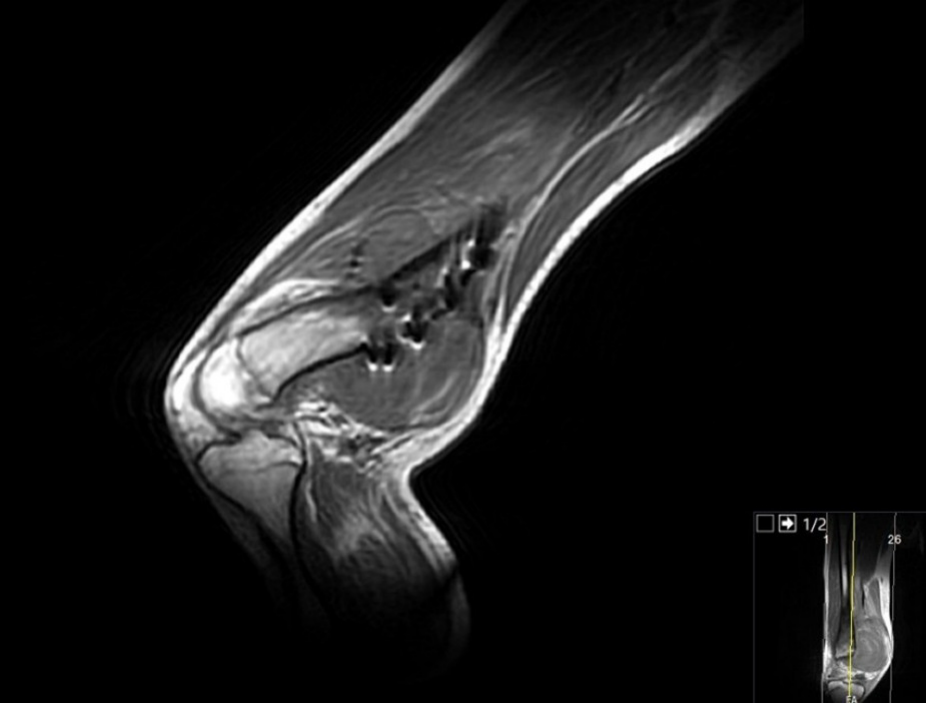
Late postoperative MRI images of the distal femur and knee joint, revealing a lobulated, heterogeneous mass extending anteromedially from the subcutaneous tissue to the bone. The lesion exhibits a mixed solid and cystic appearance with hyperintense signals on T2-weighted imaging, consistent with fluid-filled areas, and iso- to hypointense signals on T1-weighted sequences. The mass demonstrates irregular margins and appears to infiltrate surrounding soft tissues, raising concerns for a vascular or pseudoaneurysmal etiology rather than a soft tissue sarcoma. No significant perilesional edema is noted, but adjacent vascular structures, particularly the popliteal artery, are closely approximated, suggesting potential irritation or compression.

Upon arrival at our facility, a comprehensive multidisciplinary assessment was conducted by our pediatric orthopedic tumor board. The diagnostic workup included Doppler ultrasonography (Figure 3) and contrast-enhanced magnetic resonance imaging using metal artifact reduction sequences (Figure 4). However, both imaging modalities were inconclusive in definitively differentiating between a soft tissue sarcoma, hematoma, or pseudoaneurysm. Given this diagnostic uncertainty, an incisional biopsy was recommended to establish a definitive diagnosis and guide further management.

This table summarizes the patient's condition before surgery, including clinical symptoms, restricted range of motion, and imaging findings (Table 1). Key radiographic observations highlight the lobulated mass's proximity to the popliteal artery, which raised suspicion for a pseudoaneurysm.

**Table 1:** Preoperative clinical and radiographic findings.

Parameter	Details
Age and Medical History	15-year-old male with cerebral palsy, previously treated with distal femoral osteotomy.
Symptoms	Sudden and significant knee pain, localized swelling in the distal femur and knee joint.
Range of Motion (ROM)	Restricted; localized stiffness and discomfort during movement.
Imaging Modality	Doppler Ultrasound, MRI
Key Radiographic Findings	Lobulated, heterogeneous mass; cystic and solid components; close proximity to popliteal artery.

## Surgical management

The initial surgical approach was made via a medial parapatellar incision. During the surgical procedure conducted by our expert team, a significant finding was made: a mass containing hematoma was discovered nestled beneath the adductor muscles. The presence of a large hematoma confirmed the pseudoaneurysm, which was among our preliminary diagnoses. This incision, which was previously utilized for the diagnostic biopsy, was extended distally to explore the Hunter's canal and gain direct access to the lesion. Upon the release of the tourniquet that had been applied to manage blood flow, we observed active arterial bleeding originating from the damaged wall of the popliteal artery, likely due to chronic mechanical irritation by the screws. Recognizing the urgency of the situation, we promptly called for intraoperative assistance from the cardiovascular surgery team to ensure appropriate vascular repair.

To control the hemorrhage effectively, the femoral artery was clamped proximally. Subsequently, a meticulous surgical intervention was performed involving the interposition of an autologous saphenous vein graft between the intact ends of the popliteal artery, ensuring anatomical continuity and stable vascular reconstruction. The excised pseudoaneurysm cavity was sent for histopathological analysis, which confirmed the diagnosis of a pseudoaneurysm, characterized by a fibrous wall structure lacking the full arterial layers, consistent with chronic vascular injury and extravasation.

During the same session, to eliminate the source of ongoing vascular irritation, the orthopedic team re-approached the lateral distal femur using the previous surgical incision from the index procedure, which had been used for the osteotomy and plate fixation. The plate and screws were removed. Post-repair observations revealed that the most distal screws were positioned perilously close to the popliteal artery. Removal of the hardware was performed to prevent further vascular complications, especially in the context of joint motion and soft tissue tension in a spastic limb.

In the early postoperative period (first 3 days), the patient remained clinically stable. A control Doppler ultrasonography confirmed that the saphenous vein graft remained patent with no signs of thrombosis or recurrence of the pseudoaneurysm. Peripheral pulses were palpable, and no neurological deficits were observed on examination. Pain was well-controlled with standard analgesics, and the surgical wound appeared clean with no signs of infection or hematoma. At the short-term follow-up (1 month postoperatively), the patient demonstrated good recovery. The surgical incision was well-healed, and Doppler imaging continued to show full graft patency. There were no signs of recurrence or vascular compromise. The patient was mobilized with assistance, and there were no new complaints reported.

At the medium-term follow-up (3–6 months), the patient remained ambulatory with a walker and continued to participate in a structured physical therapy program. No vascular complications or hardware-related issues were noted. The patient's spasticity was stable, and his range of motion had modestly improved compared to preoperative baseline. Throughout the postoperative period, the patient demonstrated excellent adherence to medical and rehabilitation protocols. He remained compliant with his prescribed antiplatelet regimen and attended all follow-up visits as scheduled. This consistent engagement with postoperative care contributed significantly to his favorable clinical outcome.

This table provides an overview of the surgical intervention and recovery outcomes. The saphenous vein graft successfully restored vascular integrity, with imaging confirming graft patency, and follow-up revealed early mobilization and stable recovery under antiplatelet therapy (Table 2).

**Table 2:** Postoperative outcomes and follow-up.

Parameter	Details
Surgical Procedure	Saphenous vein graft interposition to repair pseudoaneurysm; plate and screw removal.
Immediate Postoperative Findings	Hematoma mass excised; arterial repair successful.
Vascular Status	Peripheral pulses palpable; restored circulation.
Imaging Confirmation	Doppler Ultrasound confirmed graft patency, no thrombosis or aneurysm recurrence.
Follow-Up Outcomes	Early mobilization; patient stable; antiplatelet regimen prescribed; regular monitoring.

## Discussion

In pediatric orthopedic cases, the dynamic nature of skeletal growth presents unique challenges in implant management. As children grow, the relative position of orthopedic implants can change due to bone elongation, remodeling, and deformity development (7). This case distinctly illustrates how distal femoral plate screws, initially positioned safely, gradually migrated posteriorly as a result of femoral deformity progression, ultimately causing irritation and injury to the popliteal artery (8). Such mechanical complications emphasize the necessity for orthopedic surgeons to anticipate the long-term impact of skeletal growth on implant positioning. Regular radiographic evaluations and clinical assessments are critical to identify early signs of implant migration or mechanical irritation, allowing for timely interventions to prevent severe complications like vascular injury (8, 9). Recent reviews emphasize that delayed removal of orthopedic implants in pediatric patients may increase the risk of complications such as stress fractures, soft tissue irritation and in rare cases vascular injury (7). Considering the patient's underlying cerebral palsy, we propose that the implant displacement may have resulted from abnormal skeletal growth dynamics and persistent spastic soft tissue forces, which are commonly seen in children with neuromotor disorders and may persist even after physeal closure.

Popliteal artery pseudoaneurysms, although infrequent, can occur as a complication following orthopedic surgeries, particularly after procedures such as distal femur osteotomy. According to Szyber P Jr et al., these instances are rare but warrant attention due to their potential implications for patient health (3). The formation of a pseudoaneurysm can arise from direct trauma to the arterial wall, or as a consequence of manipulation of the surrounding anatomical structures during surgical intervention. Despite their rarity, it is critical for clinicians to remain vigilant for such complications, especially in patients who present with delayed postoperative swelling or vascular symptoms, as highlighted by Santos-Pereira R et al. (10).

The diagnostic process for pseudoaneurysms poses significant challenges due to their presentation, which can closely resemble that of a mass lesion. This similarity can lead to misdiagnosis if not approached with caution (11). Consequently, the utilization of advanced imaging techniques becomes crucial for achieving an accurate diagnosis.

Doppler ultrasonography stands out as a valuable tool, providing real-time visualization of blood flow dynamics and enabling clinicians to assess the vascular characteristics of the lesion. This non-invasive method allows for the identification of abnormal blood flow patterns that are indicative of a pseudoaneurysm (12).

CT angiography, on the other hand, offers detailed cross-sectional images of blood vessels and surrounding tissues, facilitating the detection of vascular abnormalities with high precision. It can effectively highlight the presence of a pseudoaneurysm, delineating its size, shape, and relationship to adjacent structures. Similarly, MR angiography serves as another essential imaging modality, leveraging magnetic resonance technology to visualize blood vessels without the need for ionizing radiation. This technique provides excellent soft tissue contrast, which is particularly beneficial in distinguishing pseudoaneurysms from other lesions that may share similar imaging characteristics.

The integration of these advanced imaging modalities—Doppler ultrasonography, CT angiography, and MR angiography—plays a pivotal role in the differential diagnosis of vascular masses, ultimately guiding timely and appropriate management strategies. In our case, although CT angiography was not performed, a combination of Doppler and contrast-enhanced MR imaging using metal artifact reduction sequences provided sufficient diagnostic clarity (13).

In the pediatric population, soft tissue sarcomas represent a distinct category of rare tumors that often necessitate surgical intervention. As noted by Fletcher et al. these tumors typically manifest with symptoms such as localized pain, swelling, or the presence of a palpable mass (14). A meticulous preoperative evaluation, complemented by appropriate imaging studies, is crucial in cases where a sarcoma is suspected. This careful assessment allows for a comprehensive understanding of the tumor's characteristics and aids in formulating a strategic surgical plan that considers potential future treatment stages, as emphasized by Loeb DM et al. (15). In our clinical case, the initial postoperative evaluation under the presumption of a soft tissue sarcoma underscored the importance of a thorough and systematic assessment to guide subsequent management.

When addressing popliteal artery pseudoaneurysms, the use of autologous saphenous vein graft interposition or patchplasty has emerged as a highly effective and durable treatment option. Research by Megalopoulos A et al. indicates that this approach carries a low risk of graft occlusion or recurrence, making it a favorable alternative (16). Compared to synthetic grafts, autologous vein grafts offer significant advantages, including a reduced risk of infection and thrombosis, thereby enhancing their longevity and compatibility within the vascular system, as discussed by Sciarretta JD et al. (17). This is further supported by recent studies demonstrating favorable outcomes with autologous grafts in infrainguinal bypass surgery in challenging vascular conditions (18). Furthermore, during surgical procedures, techniques such as femoral artery clamping can be employed to minimize blood loss, thereby augmenting the overall safety and efficacy of the operation. This careful consideration of surgical strategies is paramount in optimizing patient outcomes in the context of vascular complications following orthopedic surgeries.

This case report has certain limitations inherent to its retrospective and single-case nature. First, although the diagnosis was confirmed intraoperatively and through histopathological examination, CT angiography was not performed due to clinical stability and age-related concerns, which may have provided more detailed vascular mapping. Second, the follow-up period was relatively short, and long-term functional outcomes were not assessed using standardized scoring systems. Lastly, due to the patient's minor status and neurocognitive limitations associated with cerebral palsy, a direct patient perspective could not be obtained, as recommended by CARE guidelines.

## Conclusion

This case highlights the importance of maintaining a high index of suspicion for vascular complications, particularly pseudoaneurysms, in pediatric patients presenting with sudden limb swelling and pain following orthopedic interventions. Although rare, such vascular injuries can have severe consequences if not promptly recognized and managed. In children with neuromuscular disorders like cerebral palsy, atypical growth patterns and soft tissue dynamics may alter implant positioning over time, increasing the risk of mechanical irritation and vascular compromise.

Timely diagnosis, facilitated by advanced imaging and clinical vigilance, alongside prompt multidisciplinary surgical intervention, was key to achieving a successful outcome in this case. The use of an autologous saphenous vein graft provided durable vascular reconstruction. This report underscores the need for individualized implant removal strategies and long-term follow-up in pediatric orthopedic patients, particularly when hardware is placed near major vascular structures.

## Clinical recommendations

• Ensure vascular complications are included in the differential diagnosis of atypical postoperative symptoms.
• Use Doppler, CT angiography, MARS-MRI early in suspected cases to minimize diagnostic delay.
• Coordinate care between orthopedic and vascular teams when implants are in proximity to major vessels.
• Educate referring physicians about rare but serious implant-related vascular injuries.
• Investigate long-term vascular outcomes following internal fixation in high-risk pediatric populations.

## Data Availability

The original contributions presented in the study are included in the article/Supplementary Material, further inquiries can be directed to the corresponding author.
